# Correction of malocclusion using sliding fibula osteotomy with sagittal split ramus osteotomy after mandible reconstruction

**DOI:** 10.1186/s40902-020-00266-3

**Published:** 2020-06-23

**Authors:** Dong-Hun Lee, Seong Ryoung Kim, Sam Jang, Kang-Min Ahn, Jee-Ho Lee

**Affiliations:** 1 Department of Oral and Maxillofacial Surgery, Asan Medical Center, Seoul, Republic of Korea; 2 Coreline Soft, Seoul, Republic of Korea; 3 Department of Oral and Maxillofacial Surgery, College of Medicine, Asan Medical Center, University of Ulsan, 88, Olympic-ro, Songpa-gu, Seoul, 05505 Republic of Korea

**Keywords:** Mandible reconstruction, Fibula free flap, Sagittal split ramus osteotomy

## Abstract

**Background:**

Fibula free flap mandible reconstruction is the standard procedure after wide resection of the mandible. Establishment and maintenance of normal occlusion are important in mandible reconstruction both intraoperatively and after surgery. However, scar formation on the surgical site can cause severe fibrosis and atrophy of soft tissue in the head and neck region.

**Case presentation:**

Here, we report a case of severe soft tissue atrophy that appeared along with scar formation after mandibular reconstruction through the fibular free flap procedure. This led to normal occlusion collapse after it was established, and the midline of the mandible became severely deviated to the affected side that was replaced with the fibula free flap, leading to facial asymmetry. We corrected the malocclusion with a secondary operation: a sagittal split ramus osteotomy on the unaffected side and a sliding osteotomy on the previous fibula graft. After a healing time of 3 months, implants were placed on the fibula graft for additional occlusal stability.

**Conclusion:**

We report satisfactory results from the correction of malocclusion after fibula reconstruction using sliding fibula osteotomy and sagittal split ramus osteotomy. The midline of the mandible returned to its original position and the degree of facial asymmetry was reduced. The implants reduced difficulties that the patient experienced with masticatory function.

## Background

Mandibular resection to remove oral cancer usually requires subsequent reconstructive procedures. In cases of wide resection entailing wide composite tissue defects, reconstruction with vascularized osteocutaneous free flaps is usually the most suitable option for successful clinical outcomes. Among candidate flap donor sites for mandibular reconstruction, the fibula free flap (FFF) has shown good aesthetic and functional results [1]. FFFs are suitable for both mandible reconstruction and dental implants [2]. Dental rehabilitation with implant-supported prostheses on fibula bone grafts has high survival rates and few complications [1, 3]. However, facial soft tissue contraction and atrophy of the fibula bone itself can result in unaesthetic effects and distortion of the repaired functional occlusion [4, 5].

In this case report, a patient who had oral squamous cell carcinoma developed severely distorted occlusion and facial contours after reconstructive surgery with FFF. The first reconstructive surgery used a three-dimensional (3D) printed titanium mandible and radial forearm free flap, which eventually failed and led to severe scar tissue contracture after a second reconstructive surgery. The patient underwent corrective surgery with a sliding osteotomy with a FFF and a sagittal split ramus osteotomy (SSRO) of the intact side of the mandible. Functionally stable occlusion was reestablished through the placement of implants on the fibula bone and normal facial contour was restored.

## Case presentation

An 82-year-old male with squamous cell carcinoma of the left posterior mandibular gingiva underwent a partial mandibulectomy (#35 tooth to the left mandibular angle) combined with left supraomohyoid neck dissection. Reconstruction of the composite tissue defects of the mandible was conducted with a radial forearm free flap (RFFF) for the soft tissue and a customized 3D-printed titanium block at other general hospital in Korea. The RFFF failed due to venous thrombosis, and the titanium block set in the mandibular defect was exposed out of the left submandibular area as well as the oral cavity, resulting in a wide orofacial fistula. Soft tissue reconstruction was performed using a pectoralis major myocutaneous flap to close the fistula and cover the exposed titanium block. However, the soft tissue coverage failed again and the chronic fistula was followed by severe scar contraction. When the patient was referred to the department of oral and maxillofacial surgery in Asan Medical Center 6 months after the first surgery, there was a severe infection around the exposed titanium block (Fig. 1). The presence of residual tumor was confirmed in MRI images taken after visiting our clinic. Therefore, we removed the titanium block and residual failed RFFF and performed further resection from the mesial surface of the #42 tooth to the ascending ramus of the left mandible. For the wide defect of the composite tissue defect, left side mandibular reconstruction was done with a FFF. The contour of the fibula was designed on a virtual surgery simulation and a 3D surgical template was printed (Aview(R) Modeler; Coreline Soft, Seoul, Republic of Korea). The harvested fibula was osteotomized into two parts to align with the planned contour of the reconstructed mandible. The contoured FFF was fixed by semi-rigid fixation with mini plates and mono cortical screws. After the fixation of the fibular bone, vessel anastomosis was performed, and flap perfusion was confirmed. The peroneal artery and one vena comitans were anastomosed with the facial artery and vein on the contralateral side (Fig. 2).

**Fig. 1:**
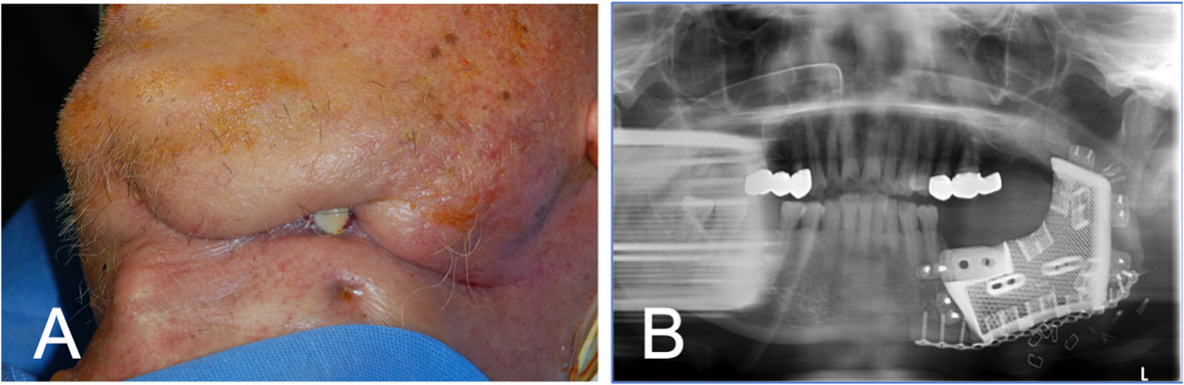
Failure of reconstruction after wide resection of mandibular composite tissue. **a** Submandibular fistula formation with chronic purulent discharge. **b** Setting state of the titanium block in the mandibular defect

**Fig. 2:**
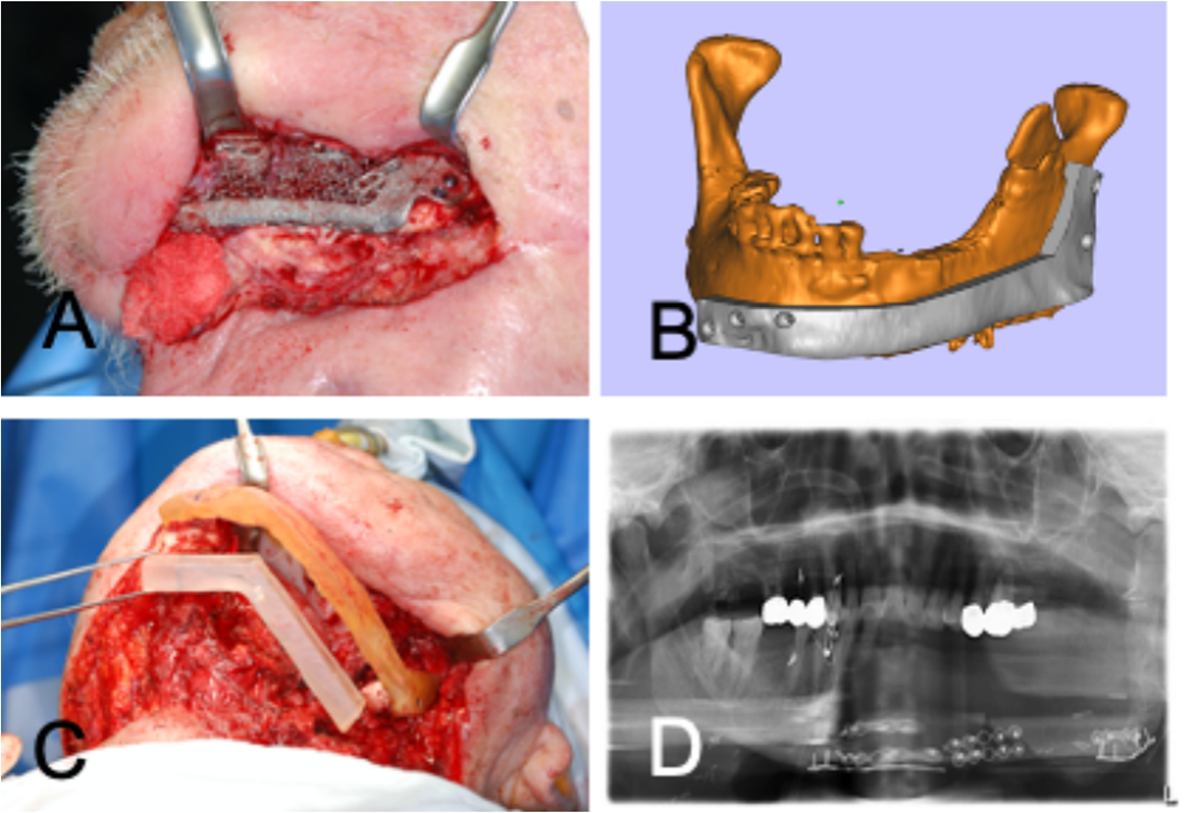
Reconstruction of mandibular composite tissue after removal of the failed graft. **a** Exposure of the 3D printed titanium block. **b** Virtual simulation surgery and design of a 3D surgical template. **c** Intraoperative application of the 3D-printed surgical template. **d** Restored mandibular occlusion after reconstructive surgery

Severe atrophy of the soft tissue at the surgical site due to scar contraction and breakage of normal occlusion, which was established intraoperatively, gradually appeared over 1 year following FFF reconstruction. Facial asymmetry developed as the midline mandible dentition severely deviated toward the side that was replaced with FFF. Therefore, an orthognathic surgical correction method was planned to solve the malocclusion and facial asymmetry caused by the scar contraction. After taking an alginate impression of the remaining dentition, a stone model was fabricated and an occlusion wafer with ideal occlusion was made. First, an arch bar was applied to the remaining teeth for intermaxillary fixation. In the mandible on the right side, the mandibular movement was obtained through SSRO. However, the virtual simulation surgery indicated that SSRO would not be sufficient to achieve normal occlusion, which was confirmed intraoperatively. Therefore, we also performed a sliding osteotomy at the anterior junction between the FFF and the #42 tooth on the mesial side. Through this, we confirmed that normal occlusion could be established, and the occlusal wafer was set between the maxilla and the mandible. After intermaxillary fixation, the segmented bones were fixed with mini plates and screws. Elastic guidance was applied to maintain the reestablished normal occlusion for 4 weeks (Fig. 3). Afterward, three fixtures of the dental implant were installed onto the FFF 3 months after surgery for stable occlusion and facial contour. The stable occlusion was maintained both at rest and during mastication after prosthodontic treatment.

**Fig. 3:**
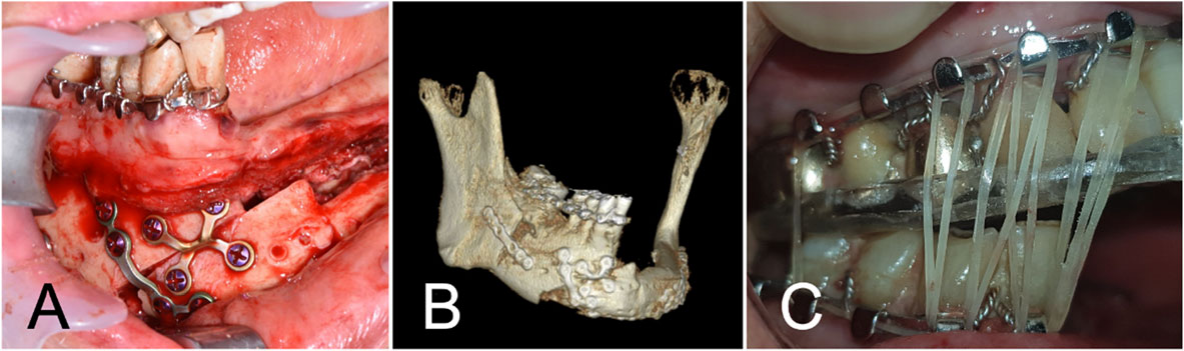
Secondary surgery for the reestablishment of occlusion and facial symmetry. **a** Sliding osteotomy at the anterior junction between FFF and #42 tooth on the mesial side. **b** 3D reconstruction image of CT scan after SSRO of the right mandible and sliding osteotomy of the anterior mandible. **c** Elastic intermaxillary fixation with occlusal wafer after restoration of normal occlusion

## Discussion

With the development of computer-aided design/computer-aided manufacturing (CAD/CAM) technology, it has been possible to reconstruct the craniomaxillofacial defects with improved preoperative planning, precise patient-specific implants (PSIs), and shorter operation times. Materials such as titanium, polyethylene, polyetheretherketone (PEEK), hydroxyapatite (HA), poly-DL-lactic acid (PDLLA), polylactide-co-glycolide acid (PLGA), and calcium phosphate are used [6]. The use of patient-specific implants avoids flap surgery to reconstruct facial defects and has the advantage of reducing the risk of harvesting from donor sites. In our case, before being referred to our institution, the patient underwent reconstruction of the left mandible bony defect using a titanium block and a soft tissue defect using a free flap from the radial forearm. However, the radial forearm free flap failed, exposing the titanium block, and a pectoralis major myocutaneous flap covering the block also failed after 2 months. According to a retrospective analysis of 881 free flaps for head and neck defect reconstruction, a history of irradiation was a statistically significant risk factor for free flap failure, while age, diabetes mellitus status, history of previous neck surgery to the anastomosis side, donor site, choice of recipient vein, use of a coupler device, and postoperative anticoagulation were not associated with free flap reconstruction outcomes [7]. However, in another study of 2846 patients with head and neck cancers, diabetes mellitus, peripheral vascular disease, renal failure, preoperative radiotherapy, and a longer duration of anesthesia were significant predictors of the occurrence of free flap failure [8]. In both studies, preoperative radiotherapy was identified as a risk factor for flap failure. Titanium mesh offers a durable repair of isolated bone defects. However, in high-risk patients with soft-tissue defects, the outcomes are significantly worse [9]. Preoperative radiotherapy, free flap coverage, and soft tissue atrophy resulted in greater odds of titanium block exposure [10]. Considering our findings and these studies, reconstructive surgery using titanium blocks and soft-tissue free flaps may not produce satisfactory outcomes, and conventional flap surgery is still necessary if the patient has been treated with preoperative radiotherapy, lacks blood vessels due to neck dissection, or if the soft tissue environment is poor. Therefore, the osteocutaneous free flap is still the gold standard for mandibular reconstruction.

Large bone defects that arise from mandibular resection can be successfully managed by fibula free flap placement [11]. Currently, the minimization of surgical errors is directly related to improved aesthetic outcomes and functional recovery [12]. In our case, to reduce the likelihood of surgical error and to reduce the operation time, a virtual surgery simulation was performed based on the preoperative CT imaging, and a rapid prototype model was prepared based on the results of the simulation. The rapid prototype model was used to determine the size of the FFF and to guide the fixation of the fibular bone to the native mandible. This drastically reduced the operation time and risk of surgical error, and the patient’s satisfaction was high.

Skin ulceration or fibrosis can develop in some patients who have undergone radiation therapy. Skin reactions may be more severe in patients with head and neck tumors than in those with tumors in other body sites [5]. Soft tissue changes due to radiation have been estimated to occur within 5 years in 1–5% of patients who have received 5500 rads, and 25–50% of patients who have received 7000 rads under certain standardized conditions [14]. In our case, severe atrophy of soft tissue and breakage of normal occlusion occurred through 1 year after fibular free flap reconstruction due to flap surgery failure occurring two times. The patient lacked sufficient soft tissue to cover the defect and vessels for microvascular reconstructive surgery. Although he did not have postoperative radiation therapy, his host tissue state was similar to that of tissue that had undergone radiation therapy. Another factor that led to occlusal changes was the atrophy of the grafted fibular bone itself. According to Ishikawa et al. [15], atrophy of the fibula graft was observed in 9.9% of the body segment and 15% of the ramal segment at 1 year after surgery elderly population. They found that fibular bone atrophy occurred mainly in the body segment in the first postoperative year. Considering the factors mentioned above, the main cause of broken postoperative normal occlusion is soft tissue atrophy due to scar contraction, but atrophy of the fibular bone that is grafted to a mandible body position also plays a role in loss of occlusion.

Several surgical methods are used to correct unexpected postoperative bone atrophy or postradiotherapy soft-tissue atrophy after mandibular reconstruction. Gennaro et al. [16] reported a new orthognathic surgery technique that improves occlusal and aesthetic outcomes in patients who underwent complex maxillomandibular reconstruction with bony free flaps. Their technique introduced the use of stepped osteotomy in the fibular reconstructed mandible and showed that this method could correct for vertical or horizontal length defects. Kim et al. [12] reported that sagittal split osteotomy of previously grafted fibula including the affected mandible ramus has several advantages. They found that the stepped osteotomy method can result in a significant interosseous gap and cause instability of the final occlusion for a long period after surgery, while the SSRO approach increased bone contact which maximized postoperative bone stability. However, in our case, SSRO approach described above was not possible because the patient’s affected mandible ramus was defective. Instead, sliding osteotomy of grafted fibula was performed and the SSRO was performed simultaneously on the unaffected mandible. This method has the advantage of achieving both vertical and horizontal movement while minimizing the interosseous gap (Fig. 4). In addition, by performing SSRO in unaffected mandible, it can be expected to increase bone contact and maximize postoperative stability than SSRO of the grafted fibula.

**Fig. 4:**
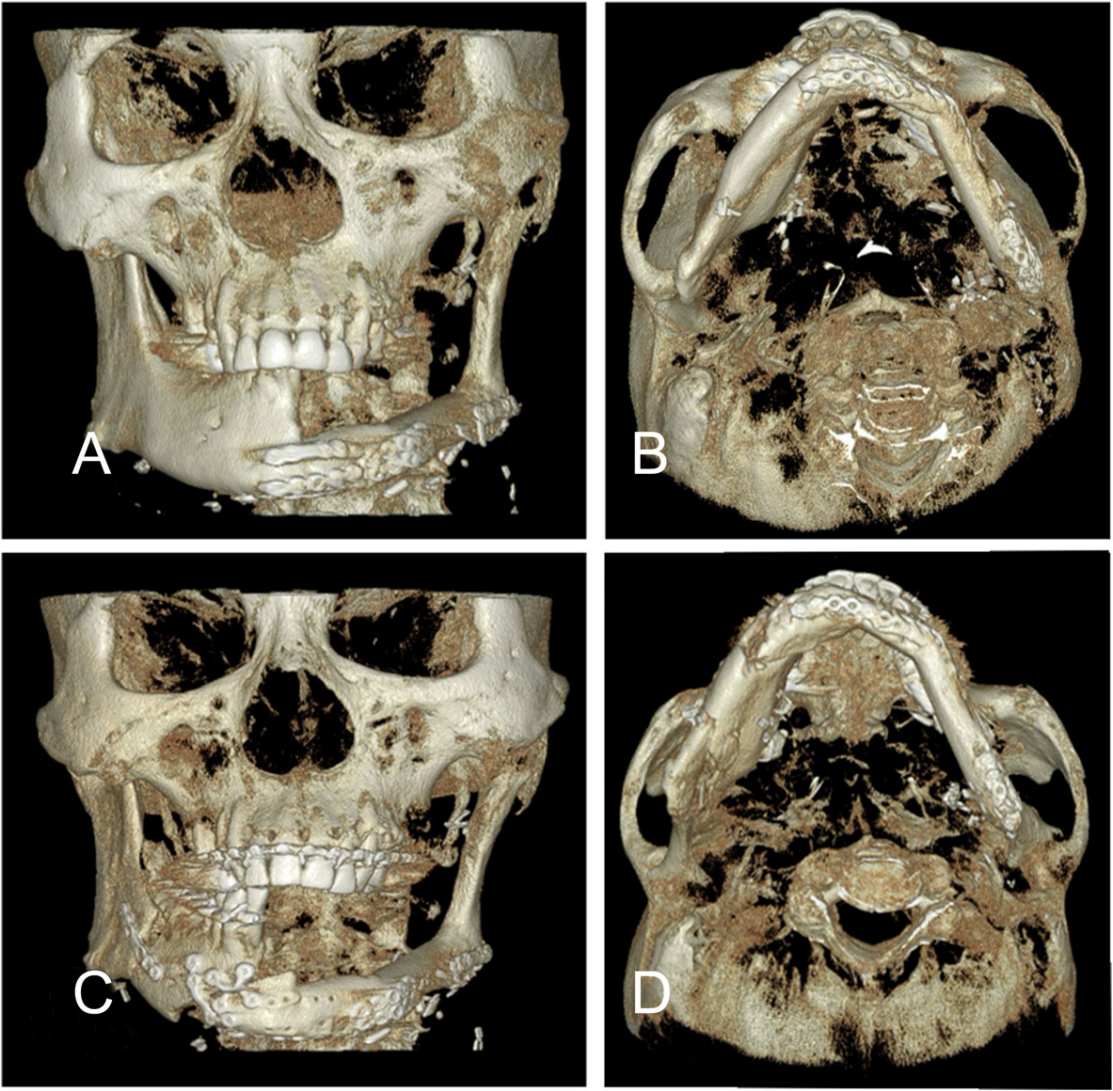
Restoration of occlusion and facial contour by sliding osteotomy combined with SSRO. **a**, **b** The patient’s mandible was moved posterolaterally to the previously operated left side, **c**, **d** but occlusion and facial contour were restored after surgery with sufficient bone contact at the junctions of the osteotomy

Longo et al. [13] proposed a new method of reconstructing the mandible angle with the SSRO of the fibula. This method reduces the risk of pedicle and endosteal vascular impairment by forming an Obwegeser-Dal Pont SSRO cutting line on the fibula bone itself. We differ greatly from this in two ways. First, Longo’s method is used for mandibular reconstruction, while our method is used when additional reoperation is needed after mandibular reconstruction. Second, in the case of Longo et al., SSRO cutting line is formed in the fibula itself, whereas in our case, SSRO is applied to the unaffected mandible.

The fibula free flap provides a consistent bone graft that allows for reliable and predictable restoration with dental implants [2]. After normal occlusion was achieved with sliding fibula osteotomy concomitant with SSRO, implants were placed in the fibula for additional occlusion stability. The implants improved masticatory function, occlusal stability, and aesthetics. Additionally, delayed placement of dental implants is associated with the prevention of fibula bone atrophy [4]. Various methods to improve the stability of dental implants placed on fibula grafts have been proposed. To compensate for the low height due to the anatomical characteristics of the fibula bone, a double-barrel type fibula graft can improve implant stability by providing bicortical anchorage of the implant [17]. Dziegielewski et al. [18] introduced a bone impacted fibular free flap technique to improve the density of fibula bones by removing bone marrow from the FFF and filling bone shavings into the marrow space. This technique shows potential for improving long-term bone density and implant stability over the FFF procedure alone.

## Conclusions

Even after successful mandibular reconstruction with a fibular bone graft, unexpected facial asymmetry and occlusal distortion may occur due to atrophy of the bone itself or as a result of soft tissue atrophy due to scar contraction. Surgical methods should be considered to solve this problem. We performed sliding fibula osteotomy with SSRO, and with this approach, normal occlusion, facial balance, and aesthetics were established. Furthermore, by placing the dental implants on the fibula bone graft, both establishment of additional occlusal stability and the restoration of the masticatory function could improve patient quality of life.

## Data Availability

All available.

## Abbreviations

FFF: Fibula free flap
3D: Three-dimensional
SSRO: Sagittal split ramus osteotomy
RFFF: Radial forearm free flap
CAD/CAM: Computer-aided design/computer-aided manufacturing
PSI: Patient-specific implants
PEEK: Polyethylene, polyetheretherketone
HA: Hydroxyapatite
PDLLA: Poly-DL-lactic acid
PLGA: Polylactide-co-glycolide acid
